# Assessment of anthropogenic pollution by monitoring occurrence and distribution of chemicals in the river Liffey in Dublin

**DOI:** 10.1007/s11356-021-14508-y

**Published:** 2021-05-25

**Authors:** Rosa Peñalver, Matthew R. Jacobs, Susan Hegarty, Fiona Regan

**Affiliations:** 1 Department of Analytical Chemistry, Faculty of Chemistry, University of Murcia, Murcia, Spain; 2 School of Chemical Sciences, Dublin City University, Glasnevin, Dublin, 9 Ireland; 3 DCU Water Institute, Dublin City University, Glasnevin, Dublin, Ireland; 4 School of History and Geography, Dublin City University, St Patrick’s Campus, Drumcondra, Ireland

**Keywords:** Distribution, Liffey river, Gas chromatography-mass spectrometry, Phthalates, Chlorophenols, Nitro-aromatic derivatives

## Abstract

This paper evaluates for the first time the spatial distribution of a wide group of organic (phthalates, nitro, aliphatic, halogen, aromatic, phenol and amino compounds) and inorganic pollutants along the Liffey river in Dublin city. The work takes into account the effect of short-term weather conditions on the occurrence of these contaminants. The results showed that rainfall conditions affect the levels of pollutants along the river in the days following a rainfall event. In addition, the tributaries entering the river Liffey were not found to impact its water quality. In relation to organic pollutants, 2,4,6-trichlorophenol, 2-nitrophenol and phthalate compounds were found in many water samples between concentrations of 0.21 and 2.17 μg L^−1^. On the other hand, dimethyl phthalate was present in certain samples at levels around 100 μg L^−1^. The levels of these contaminants in the river were lower than the toxicity values reported in the literature. Regarding inorganic pollutants, nitrates were detected from 0.59 to 6.81 mg L^−1^ increasing from upstream to downstream. Based on the chemical nature and applications of detected pollutants, the river contamination can be mainly related to agricultural, industrial activities as well as diffuse urban contributions. These vary with location within a short distance and have the potential to impact aquatic biodiversity as the chemical composition changes with rainfall events.

**Supplementary Information:**

The online version contains supplementary material available at 10.1007/s11356-021-14508-y.

## Introduction

Environmental agencies worldwide monitor river waters for the presence of inorganic and organic contaminants to determine their quality status (Boi et al. 2016) as part of Water Framework Directive (WFD) monitoring (European Commission 2000). With the Green Deal headline objective of zero pollution (European Commission 2019), there is a huge challenge in managing the release and effects of chemicals entering our waterways. This chemical strategy for sustainability is designed to better protect citizens and the environment against hazardous chemicals by improving monitoring, reporting on pollution levels and remediating at risk water bodies. In this context, this paper is a study of organic and inorganic pollutant occurrence in a river flowing through a capital city. Anthropogenic sources of pollutants in river water are derived from three main sectors including agricultural land use, industrial operations (such as food processing, manufacturing, waste management) and urban domestic contributions. Conventional wastewater treatment plants (WWTP) and other waste management infrastructures face a significant challenge in removing many pollutants which may be released to river water sources (Happonen et al. 2016), often due to aging infrastructure or management issues. The many anthropogenic activities contribute a diverse range of pollutants to aquatic systems either as a result of the use of chemicals, their accidental release during transportation/storage, as leachates from industrial operations, waste processing sites and landfills or as degradation products of other chemicals (Baker 2005; Yusof et al. 2009).

Among the organic pollutants, phenols, chlorophenols (CPs), nitrophenols (NPs) and phthalate esters (PAEs) are of particular concern. CPs and NPs are important intermediates in the production of pesticides, preservatives and dyes (Faludi et al. 2015). CPs are toxic and carcinogenic compounds that have been shown to bioaccumulate, and they are resistant to degradation. Therefore, their usage has been restricted (Xu et al. 2017). NPs are used in the manufacture of adhesives, explosives, paints, pharmaceutical and pesticides products and are also toxic (Pastor-Belda et al. 2018). Phthalates are widely used as plasticizers in household products, detergents, flame retardants, plastics, cosmetics, adhesives and medical devices (Net et al. 2015). They may accumulate in surface waters as a result of their release during production of plastics, leaching from plastics containing phthalates in consumer products, or by inefficient removal by wastewater treatment plants (Net et al. 2014). Some phthalates such as, bis(2-ethylhexyl) phthalate are regarded as priority pollutants by the EU Directive 2008/105/EC (European Commission 2008) due to their suspected carcinogenic and endocrine disruption actions.

River water quality depends not only on the presence of organic contaminants but also on the presence of inorganic species or nutrients. The release of compounds such as nitrates and phosphates to rivers can cause lead to eutrophication which has significant negative impacts on river and surface water health (Zhao et al. 2019). Hydrological conditions, subsoil geology and seasonal variation can influence levels of inorganic species (nitrate and sulfate) in rivers which can be monitored by the usage of isotopic techniques (Rock and Mayer 2006). Sulfates and nitrates are common components of fertilizers used in agriculture which can contribute to excessive levels of these species in rivers.

The objective of this work is to evaluate the occurrence, levels and distribution of toxic pollutants and inorganic species in the Liffey river in Dublin city. The compositions of the organic and inorganic contaminants along the river on different sampling days were determined by gas chromatography-mass spectrometry (GC-MS) after a preconcentration and clean up step by solid phase extraction (SPE). Ion chromatography (IC) was used to evaluate the levels of common inorganic anions including chloride, nitrite, nitrate, phosphate and sulfate as these species are linked with river health.

The aim of this work is to demonstrate the occurrence of chemical substances (organic and inorganic) in a river flowing through agricultural land and into a large urban area, using sample collection over a short timeframe to demonstrate variability in the event of heavy rainfall. The analysis is targeted in order to investigate the variability of a range of potential pollutants along a river course that has multiple diffuse and point sources. The choice of chemicals the authors believe makes this study transferable to any similar river exposed to urban and rural activities. This is therefore a useful assessment of the health of a river and potential for remediation under the Green Deal objectives towards ‘zero pollution’.

## Material and methods

### Site description

**Fig. 1:**
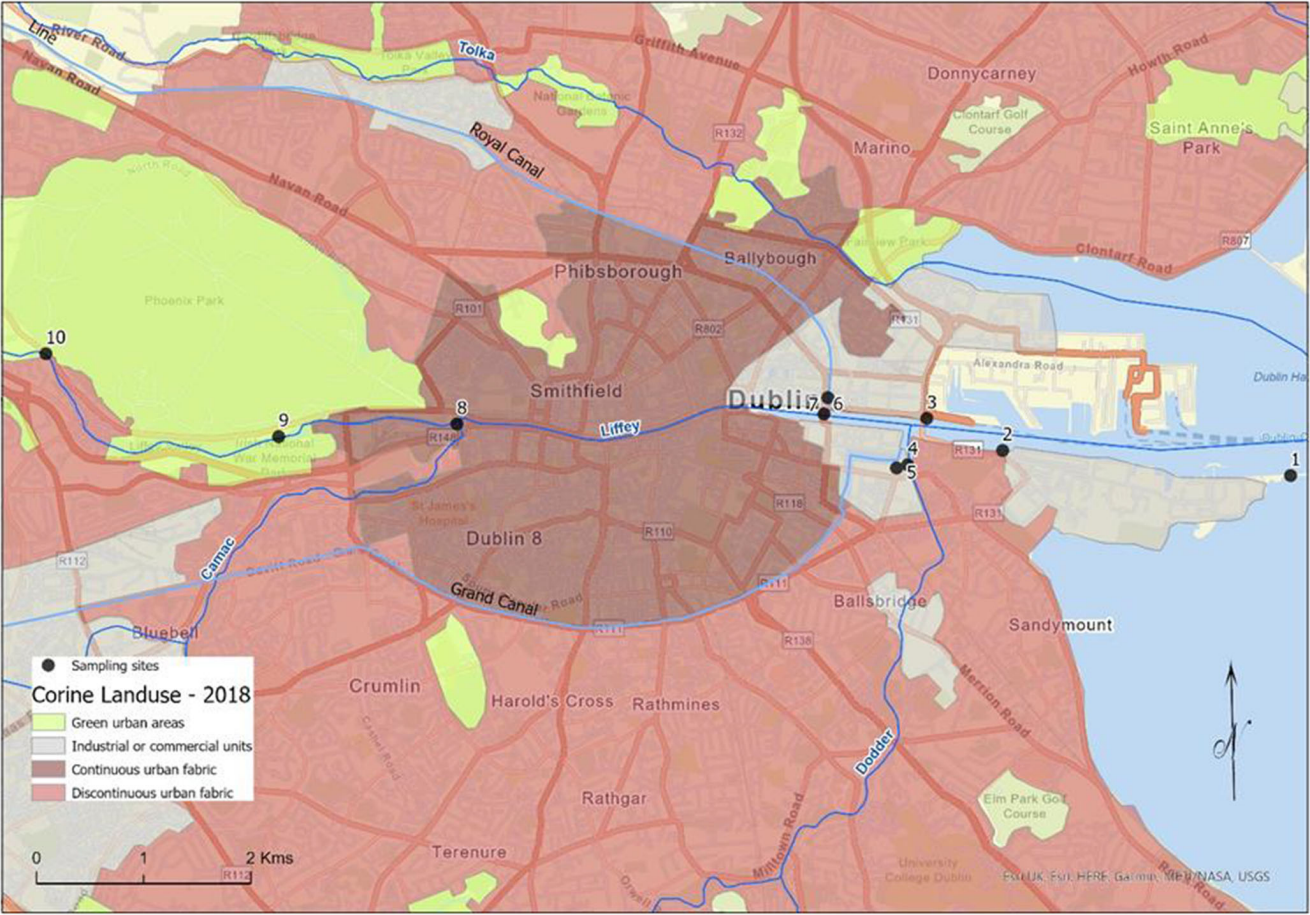
Map showing the sampling sites (1–10) along the river Liffey. Map shows urban landcover in the study area, as well as the water courses that run through the city.

The river Liffey rises between Kippure and Tonduff in the Wicklow mountains and flows for approximately 125 km through counties Wicklow, Kildare and Dublin before entering the Irish Sea at its mouth at the midpoint of Dublin Bay. The Liffey is a controlled river that has a regulated flow for the final 80 km of its journey, due to three hydroelectric power stations, at Poulaphouca, Golden Falls and Leixlip, as well as a number of minor private installations and historical weirs. Major reservoir facilities also exist at Poulaphouca where significant waterfalls there and at Golden Falls were flooded during reservoir construction. The annual average flow rate of the Liffey river at Leixlip is approximately 2.35 m^3^ s^−1^. Low flow conditions are maintained at 2.00 m^3^ s^−1^. The Liffey is tidal from the weir at Islandbridge (at sample site 9 on Fig. 1) to the mouth of the river (8.5 km), in the reach of the river that flows through Dublin city centre. Eight of the sampling sites for this paper are located within this tidal area, with sites 9 and 10 within the freshwater section of the river. The mean tidal range is 2.75 metres (Bedri et al. 2013).

The Liffey is classified as a nutrient sensitive water body and is considered to be at high risk from diffuse pollution through groundwater and urban run-off and from point sources located within its catchment (O'Higgins and Wilson 2005; Brooks et al. 2016; EPA 2018).

### Reagents and solutions

An internal standard (mixture of Acenaphthene-*d*_*10*_, Chrysene-*d*_*12*_ 1,4-Dichlorobenzene-*d*_*4*_ Naphthalene-*d*_*8*_, Perylene-*d*_*12*_, Phenanthrene-*d*_*10*_) at 4000 μg mL^−1^ for each component, Base Neutral Surrogate at 5000 μg mL^−1^ (mix of 2-Fluorobiphenyl, 2-Fluorophenol, Nitrobenzene-*d*_*5*_, Phenol-*d*_*5*_, *p*-Terphenyl-d_14_, 2,4,6-Tribromophenol) and 8270 Mega Mix [Supplementary Information (Table S1)] 1 mL at 1000 μg mL^−1^ in dichloromethane were purchase from Restek (Bellefonte, PA, USA). Working solutions at 1 μg mL^−1^ were freshly prepared by diluting the stock solutions in dichloromethane. All the solutions were stored at 4 °C in amber vials in the dark. Sodium carbonate (#791768), sodium hydrogen carbonate (#1063290500), sodium fluoride (#201154), sodium chloride (#43320-9), sodium chlorate (#244147), sodium nitrate (#1065370500), sodium phosphate (#S9638) and sodium sulfate (#239313) were purchased from Sigma Aldrich, Ireland. Sodium nitrite (#424350020) was purchased from ACROS organics, Ireland. Ion chromatography eluent contained 4.5 mM of sodium carbonate and 1.4 mM of sodium hydrogen carbonate dissolved in deionized water (18.2 MΩ cm^−1^, ELGA Pure Lab Ultra, VWR, Dublin, Ireland).

Oasis hydrophilic lipid balance (HLB) solid phase extraction (SPE) cartridges with a 3 mL volume, 60 mg bed mass and 30 μm particle size were purchased from Waters Chromatography (Dublin, Ireland), and a 12 port SPE vacuum manifold (Phenomenex, Ireland) was used for SPE sample preparation. Pesticide grade dichloromethane (Fisher Scientific, Dublin, Ireland), MS grade acetonitrile (Sigma Aldrich, Arklow, Ireland) and deionized water were used for the solid phase preconcentration procedure. Ultrapure helium (Air Products, Dublin, Ireland) was used as the carrier gas in the GC system.

### Instrumentation and software

An Agilent 6890N gas chromatograph (Agilent Technologies, Cork, Ireland) equipped with a split/splitless injector (Agilent 7683 series automated liquid sampler) and connected to an Agilent 5973 Network Mass Selective Detector was used for analysis of organics in river water samples. A weakly polar VF-5ms column coated with 5% phenyl/95% dimethyl-polysiloxane polymer equivalent, 30 m × 250 μm I.D × 0.25 μm *d*_*f*_ (Agilent) was used for separation as suggested by EPA method 8270 (Agency 2015). The inlet temperature was set to 270 °C and operated in the pulsed splitless mode with a head pressure of 30 psi held for 0.6 min during sample injection. A volume of 1 μL was injected for all samples. The carrier gas was helium which was set to a constant flow rate of 1.2 mL min^−1^. The GC oven was programmed from an initial temperature of 40 °C which was held for 1 min, and then ramped at 25 °C min^−1^ to 280 °C, followed by ramping at a slower rate of 5 °C min^−1^ to a final temperature of 320 °C which was held for 1 min. The total separation time was 19.6 min. The MS transfer line was set to a temperature of 280 °C. The mass spectrometer was operated with a source temperature of 180 °C and a quadrupole temperature of 150 °C and a detector voltage of 70 eV. Data was acquired from 2.2 to 19.6 min from 35 to 550 *m/z* at a scan rate of 5.36 scans s^−1^. Compound identities were determined using authentic standards where possible and library searching with NIST 98 Mass Spectral Library (Agilent) and further confirmed using retention index matching.

Ion chromatography was performed on a Dionex ICS-3000 instrument (Thermo Fisher Scientific, Ireland) equipped with a Dionex AS autosampler (Thermo Fisher Scientific). Separations were carried out using a Dionex IonPac AS-22 column (4 × 250 mm, Thermo Fisher Scientific) with a 10-μL sample injection loop. Separation was carried out isocratically using an eluent composed of 4.5 mM sodium carbonate with 1.4 mM sodium hydrogen carbonate dissolved in deionized ultrapure water (resistivity 18.2 MΩ cm^−1^), with a flow rate of 1.2 mL min^−1^ and a separation time of 15 min. A Dionex AERS-500 4 mm anion self-regenerating suppressor (Thermo Fisher Scientific) with an operating current of 31 mA was used for suppressing background eluent conductivity. Peak detection was performed using a Dionex conductivity cell with a 10-mm path length (Thermo Fisher Scientific) that was operated at a data collection rate of 5 Hz with a rise time of 0.5 s. The column and detector compartments were maintained at 30 °C, while the detector cell was maintained at 35 °C. Data was collected and processed using Chromeleon 7 Chromatography Data system (version 7.2 SR4 8179, Thermo Fisher Scientific, Ireland).

### Sample extraction and analytical procedure

A 100 mL volume of river water sample was spiked with a concentration of 1 mg L^−1^ of standard mixture prior to extraction. SPE cartridges were conditioned with 3 mL of dichloromethane, followed by 3 mL of acetonitrile and finally 3 mL of deionized water. The sample was then loaded onto the conditioned SPE cartridge using vacuum assistance. Cartridges were then dried using vacuum application for 30 min, after which 3 mL of dichloromethane was used to elute the SPE cartridge. The volume of dichloromethane was reduced to 0.5 mL under a stream of nitrogen gas, and a mixture of internal standards was then added prior to GC-MS analysis. Triplicate injections were carried out in the GC-MS system. Method blanks were performed using 100 mL deionized water in place of 100 mL of river water.

### Analytical characteristics of the method

Calibration curves were obtained using standard solutions prepared at six different concentrations, ranging from 0.01 to 5 mg L^−1^ (with the IS mixture at 1 mg L^−1^), applied to the GC-MS single ion monitoring (SIM) method. Supplementary Information (Table S1) shows the selected ions of each compound that were monitored during the SIM method. Correlation coefficients higher than 0.99 were obtained for all compounds.

The sensitivity of the method was evaluated through the values of limits of detection (LODs) and quantification (LOQs) which were calculated by signal to noise ratio of 3 and 10, respectively [Supplementary Information (Table S1)]. LODs ranged from 0.025 to 0.73 μg L^−1^ and LOQs were in the range of 0.083 to 2.43 μg L^−1^. To evaluate the precision of the method, a river water sample was submitted to three consecutive SPE-GC-MS analyses, obtaining the average relative standard deviation (RSD). The RSD values were in the range 0.007–17.1% for all the compounds reported. Spiking experiments were carried out at 1 mg L^−1^ to calculate the recovery values for all the compounds [Supplementary Information (Fig. S1)]. From the total of compounds, thirty-four pollutants have recovery values over 50%.

### Site description and sample collection

Figure 1 shows the sampling sites and illustrates the types of activities and land use along the river course. Ten sampling sites were selected along the Liffey river from Dublin Bay area (S1) to freshwater upstream (S10) (Fig. 1). A total of 80 samples were collected on 4 separate sampling dates in the period between August 1st (Day 1) August 17th (Day 2), August 23rd (Day 3) and September 11th (Day 4) along the Liffey river (Dublin, Ireland). Rainfall measurements corresponding to the total rainfall for two days prior to each sample collection where 22.9 mm, 7.3 mm, 0.1 mm and 2.2 mm of rainfall for days 1 to 4, respectively. These values are obtained from the Phoenix Park weather station, located in the parkland to the north of S9 and S10 and published by Met Éireann (Éierann 2019). Duplicate water samples were taken at each site using 500 mL high density polyethylene plastic bottles. The bottles were pre-rinsed with ultrapure water and the river water sample before the sample collection. All the collected samples were stored at 4 °C prior to extraction.

The sites were selected for sampling at locations where there are inputs to the river Liffey (Fig. 1) — the river Camac (site 8), the river Dodder (site 4), the Grand Canal (site 5), the Royal Canal (site 7), as well as other sites which act as a baseline for the study.

### Data analysis

Data analysis was performed using R version 3.6.2 (2019-12-12) and RStudio version 1.2.5033. Plots and data summaries were prepared using dplyr (version 0.8.5) and ggplot2 (version 3.3.0). The sample sites were mapped using ArcGIS Pro (version 2.6.2). The data was summarized into compound groups based on their chemical composition [Supplementary information (Table S2)]. This enabled the compound groups to be plotted as bar charts within the GIS, to show the different concentrations of compound groups present within the samples on each of the dates. The differences of concentrations were thus highlighted on both spatial and temporal scales.

## Results and discussion

### Organic pollutant content in Liffey river

**Fig. 2:**
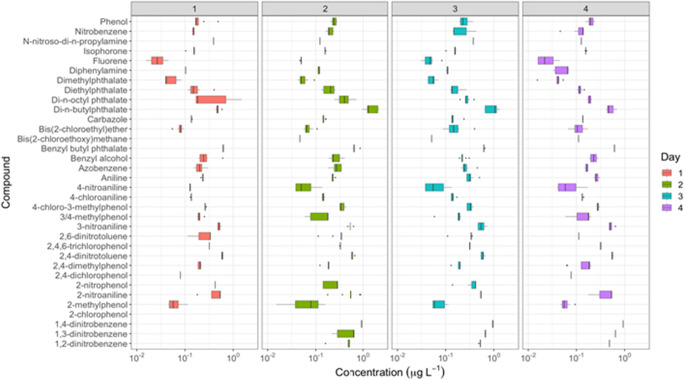
Concentrations (μg L^−1^) of target chemicals measured in water samples on each of the four sampling days (1–4). There were two outliers for dimethylphthalate; Day 2 for sampling locations 4 (105.01 ug/L) and 8 (48.09 ug/L). N = 3 which have not been plotted for reasons of clarity

A standard mixture of organic analytes was chosen to act as an indicator of water quality along the course of a river. From a total of eighty-two compounds included in the standard mixture, thirty-four have shown recovery values higher than 50% [Supplementary Information (Fig. S1)]. The specific SPE conditions described in section 2.4 as well as the usage of hydrophilic lipid balance (HLB) cartridges made the extraction step selective for certain chemical compounds. As can be observed in [Supplementary Information (Fig. S1)] phthalates, nitro-aromatic and phenol compounds showed good recovery values. However, PAHs showed low recovery values except for carbazole and fluorene. Figure 2 shows the results of pollutant occurrence and concentration from samples collected on the four sampling days. Certain pollutant concentrations vary over two orders of magnitude, while most occur in a very narrow range and are below reported acute toxicity levels.

**Fig. 3:**
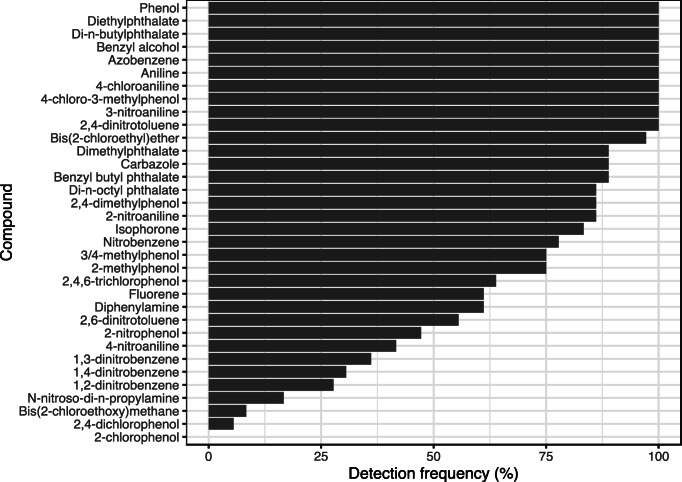
Percentage of positive samples of the targeted compounds detected in the samples during the study period

From the analysis of 80 samples, it was found that 10 of the target species were present in all samples (Fig. 3). Those present include phthalate and phenolic species with log K_ow_ values in the range 1.3–4.5. It was found that di-chlorophenols were rarely detected in the Liffey samples and 2-chlorophenol was not found in any sample. However, 2,4,6-trichlorophenol isomer was found in many river samples (greater than in 60% samples analysed) with an average concentration of 0.21 μg L^−1^. Although the specie 2,4,6-trichlorophenol is a carcinogenic substance listed in the Fourteenth Report on Carcinogens (U.S. National Toxicology 2016), the concentration levels found in the river were much lower than the toxicity levels of this compound to different aquatic organism. The toxicity reported values were 3,198, 5,470 and 3,732 μg L^−1^ to Chlorella, Daphnia and Tilapia, respectively. On the other hand, this compound has been quantified by other authors (Wang et al. 2012) in samples from the Pearl River in China where levels between 0.021 and 0.205 μg L^−1^ were detected. Alkylphenol compounds, 2,4-dimethylphenol and methyl phenol, were present in the samples (> 70%) at an average concentration of 0.15 and 0.12 μg L^−1^, respectively.

In the case of nitro-aromatic compounds, the 2-NP isomer was present in several river Liffey samples (in half of the total samples) between 0.045 and 0.43 μg L^−1^. This compound is reported to pose significant health risks since it is highly toxic to microorganisms, mammals and anaerobic bacteria. The nitro group may be easily reduced by enzymes to a nitro anion radical, and subsequently nitroso and hydroxyl-amine derivatives which have mutagenic and carcinogenic effects (Md. et al. 2020).It is important to highlight that the levels detected in the river are lower than the acute lethal toxicity levels reported by Y. Jui-Hung et al. (2002) for this compound to two different aquatic organisms. The 2-NP toxicity values were 289 and 565 μg L^−1^ to Daphnia and Carp, respectively. NPs isomers have been detected by other authors and associated with leaching from irrigation waters. The obtained values are in the same range as selected samples from the river Liffey (approximately 0.2 μg L^−1^) (Pastor-Belda et al. 2018). Dinitrobenzene and dinitrotoluene compounds were found with 1,4-dinitrobenzene at the highest concentration close to 1 μg L^−1^. This measured value is nine times lower than the acute toxicity level reported by Y. Jui-Hung et al. (2002) for this compound to aquatic organisms (9 μg L^−1^). It is interesting to highlight that 2,4-dinitrotoluene was present in all the samples as shown in Fig. 3. These different isomers are considered carcinogenic compounds (Forschungsgemeinschaft 2018). However, similar to the other nitro-aromatic compounds, the 2,4-dinitrotoluene was present as a much lower level than the toxicity one reported in the literature by Y. Jui-Hung et al. (2002) (around 100 μg L^−1^). However, synergistic effects or chronic long-term effects of any of these pollutants in the ecosystem are unknown.

In relation to aliphatic compounds, the presence of bis(2-chloroethyl) ether was observed in almost all the samples along the river during the four sampling days with an average concentration of 0.11 μg L^−1^.

Five phthalate ester compounds were quantified in this study. Phthalates are used as plasticizers and in products such as insect repellent (e.g. dimethyl phthalate). These compounds were frequently detected in the Liffey river samples (Fig. 3). The levels of these pollutants ranged from 0.025 to 2.17 μg L^−1^ in the different samples except the dimethyl phthalate which was present at levels around 125 μg L^−1^ in certain samples. Although, the detected level of dimethyl phthalate is significantly higher than values reported for other European rivers such as the Somme River in France where the total concentration of six phthalate compounds was approximately 20 μg L^−1^ (Net et al. 2014), the level detected in the Liffey river for this compound is much lower than reported toxicity levels (Staples et al. 1997). Meanwhile, butyl benzyl phthalate and di-*n*-octyl phthalate were detected in the river Liffey samples, while they were not found in the Somme River. The measured levels of many these pollutants in the Liffey river are below acute toxicity values.

A. Pistocchi et al. (2019) have identified emission patterns and dissipation half-lives of some priority substances. These authors developed a model that determined the main factors to be considered to describe chemical pollution at a European scale. Similar families of pollutants were evaluated in this study, and it was observed that the main loads to European seas were phthalate esters followed by chlorophenol-type compounds. This river Liffey study showed similar results with phthalate compound occurrence being most prevalent together with the occurrence of 2,4,6-trichlorophenol and nitro-aromatic compounds as discussed above (Fig. 3).

The average level of di-*n*-butyl phthalate (0.87 μg L^−1^) was lower than the environmental risk limits specified by Van Wezel et al. (2000) who define an limit 10 μg L^−1^ for di-*n*-butyl phthalate in river waters is while the levels observed in the Liffey samples was in the range of 0.42 to 2.17 μg L^−1^. Figure 3 shows that of the thirty-four targeted pollutants found in the samples, the PAEs were the most frequently detected species. On the other hand, polycyclic aromatic hydrocarbons (PAHs) were also found to be present in a low percentage of samples within the study area. The main source of these compounds is related to incomplete combustion processes, either naturally or anthropogenically derived (Abdel-Shafy and Mansour 2016).

Considering the main targeted pollutants present in the river and their applications, it can be highlighted that the main sources of contamination can be linked to agricultural and industrial activities as well as contamination from urban residues as the river flows from agricultural to urban areas.

### Organic pollutant groups in Liffey river on the four sampling days

The contaminants were compiled into groups according to their chemical structures [Supplementary information (Table S2)]. These groups are phthalates, aromatic and aliphatic compounds.

**Fig. 4:**
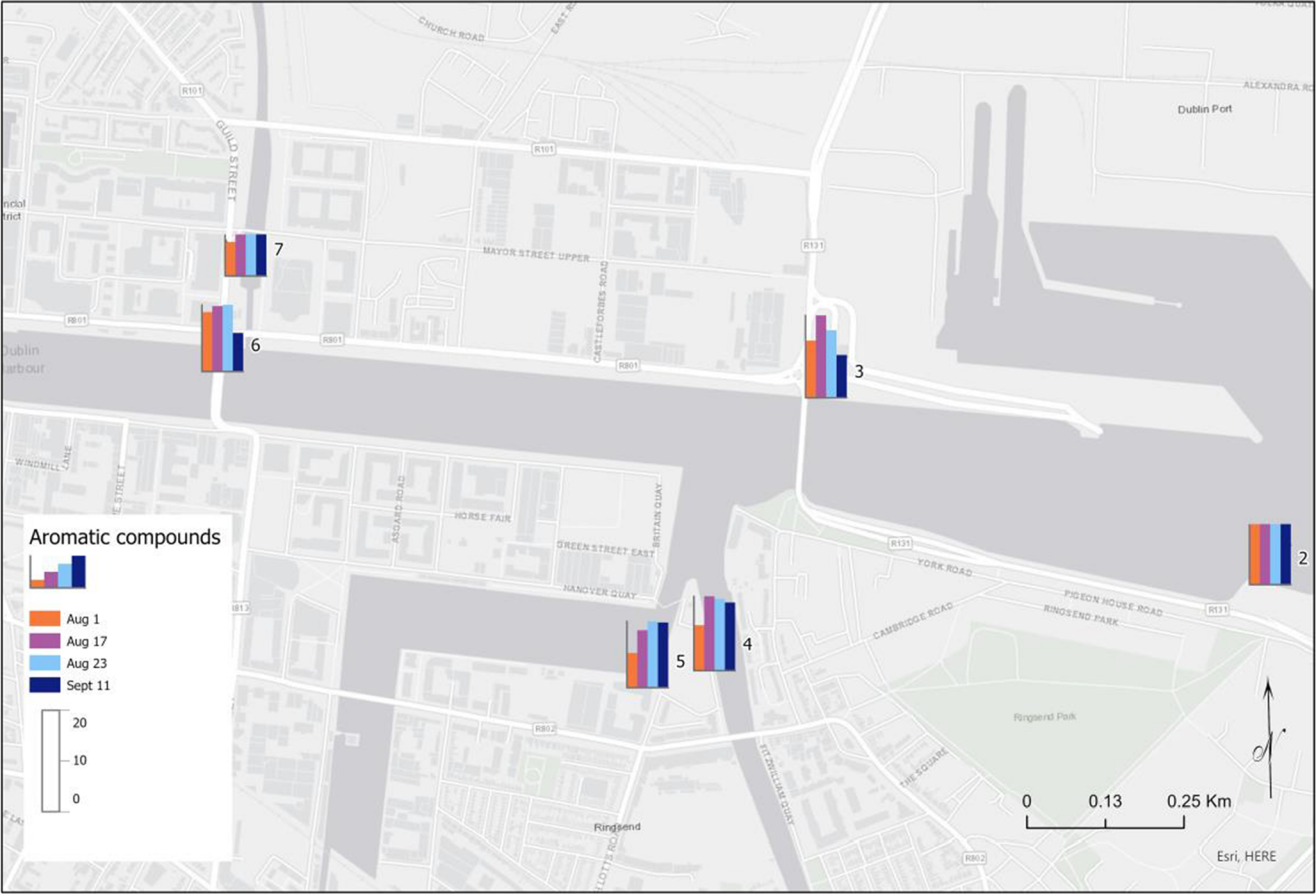
Levels of phthalate compounds near the mouth of the river on four sampling days, highlighting the impact of flows into the Liffey

**Fig. 5:**
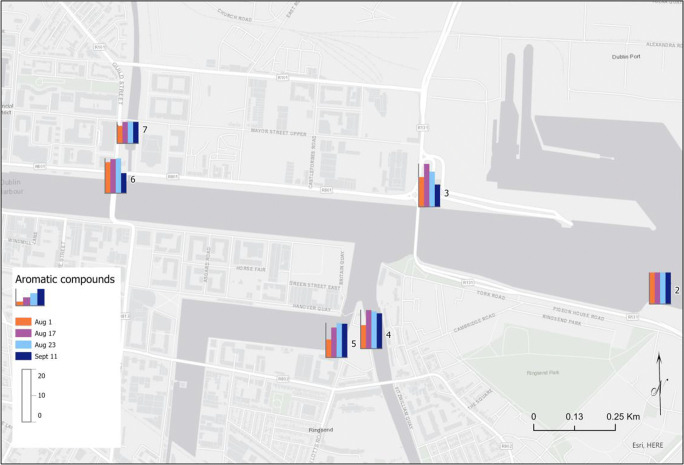
Levels of aromatic compounds, excluding phthalates, near the mouth of the river on four sampling days

**Fig. 6:**
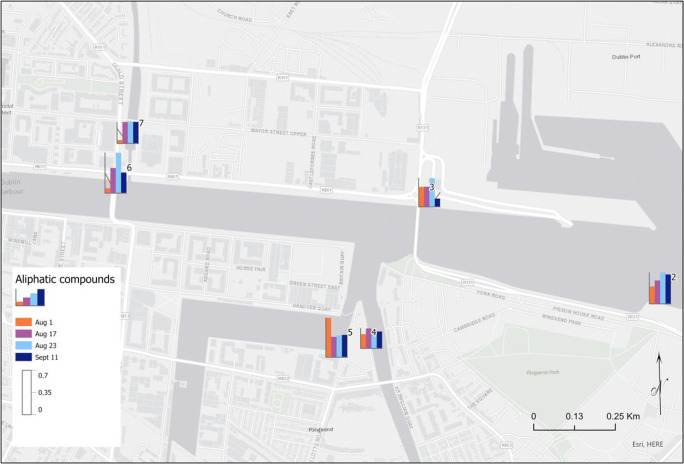
Levels of aliphatic compounds near the mouth of the river on four sampling days

Figures 4, 5 and 6 show the levels of the different groups of contaminants in the sampling sites 2 to 7, on each of the sampling days. There was a trend where most pollutant groups showed lower levels at the first sampling day along the river except for aliphatic compounds measured at site 5 where the levels of these compounds on that sampling Day 1 were higher than the other sampling dates. Considering the heavy rainfall (> 20 mm) event preceding the 2 days before the sample collected on Day 1, concentrations were expected to decrease where runoff may dilute dissolved pollutants. It is noteworthy that site 5 is within the Grand Canal basin. The basin (and the canal in general) is not as responsive to rainfall events as flow is controlled along the length of the canal by locks and canal walls. Several conclusions have been made by different authors regarding the effect of seasonal effects on pollutant concentration in surface waters. For example, the study of Heo et al. (2020) indicated that rainfall events typically dilute PAE concentrations in the seawater of the beaches. Huang et al. (2015) reported that the effect of rainfall on the PAH concentrations in the Pearl River Estuary was insignificant due to the different contamination sources for these types of pollutants. It has been concluded that the input of contaminants into the surface water environment depends on the physicochemical characteristics of the environment (soils, pH, rainfall level) and each pollutants’ respective properties. Therefore, it is difficult to generalise about the conclusions (Heo 2020; Moreno-González et al. 2013a; Moreno-González et al. 2013b).

On the other hand, in the case of phthalates and aromatic compounds, it was observed that the highest levels in most of the sampling sites were detected on Day 2 of sampling prior to which there was little rainfall. An outlier was detected during measurement of phthalates where high levels were found for the river Dodder during the Day 2 of the study at site 4. On this day, flood alleviation works were being carried out on the river Dodder, 1.5 km upstream of site 4, which may have led to an increase in the levels of phthalates detected.

The Royal and Grand canals contribute to the river Liffey using river locks ensuring that backflow into these contributing waterways is not possible.

**Fig. 7:**
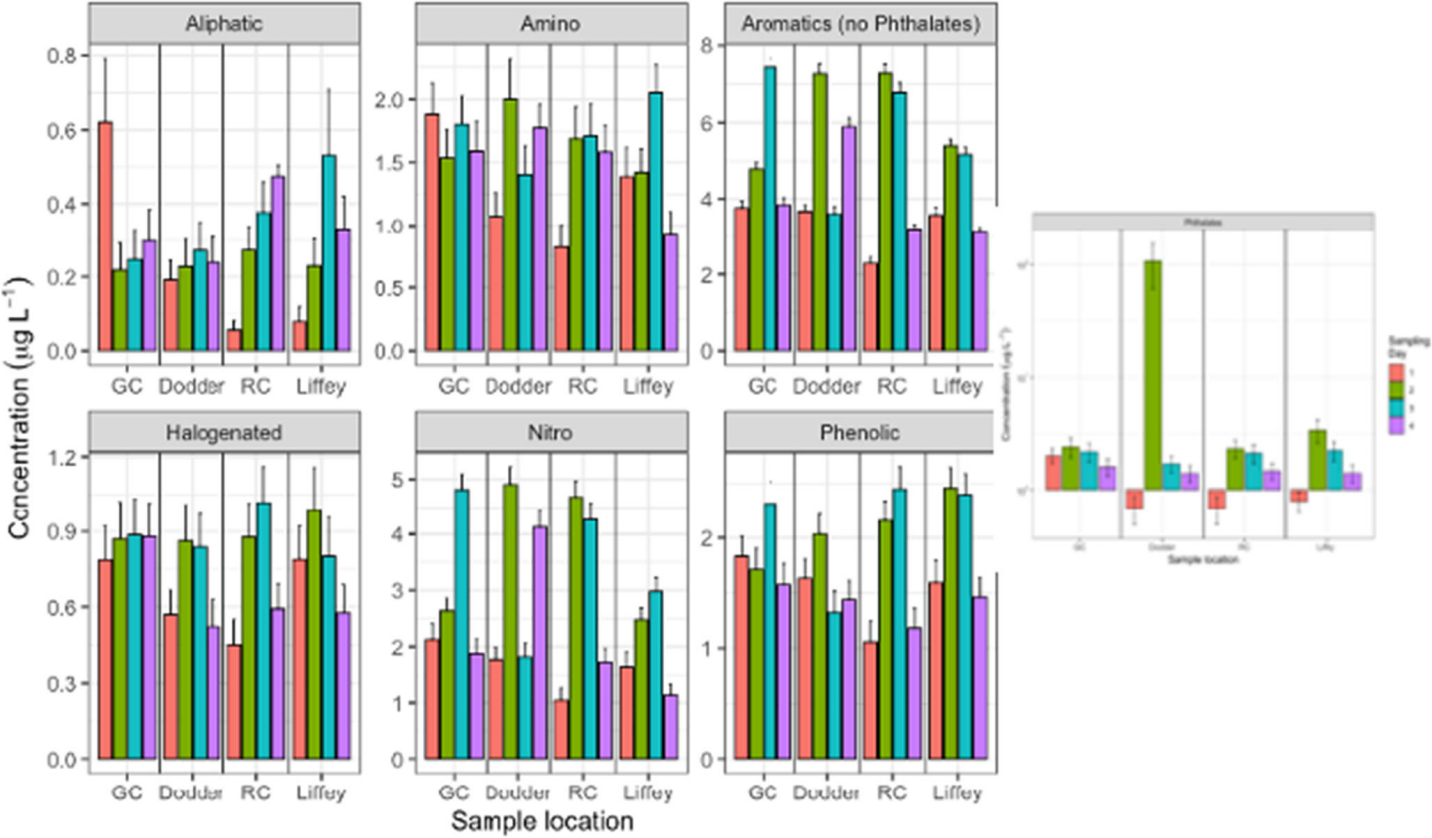
Comparison of grouped organic compound concentration based on functional group classification. Levels of species are shown for the Liffey river (Liffey, site 6), the Grand canal (GC, site 5), the Royal canal (RC, site 7) and the Dodder river (site 4) for each of the four sampling dates. Error bars calculated for n = 3

In the case of aromatic and nitro compounds, the levels of these pollutants in the Liffey (site 6) were, in general, lower than in the canals and Dodder river which connect to the Liffey. It may be explained by a dilution effect due to the water input from the canals to the river for these compounds. However, for the other groups of pollutants, halogenated, phenolic, amine and phthalates, there were not any tendency observed. As a remark, the flood alleviation works carried out the sampling Day 2 of phthalates as shown in Fig. 7.

### Inorganic pollutants in Liffey river

Natural waters contain a range of chemicals at levels consistent with and influenced by their surrounding environments. Where levels increase above these background levels they are seen as pollutants. Unlike the organic pollutants studied, many nutrients occur naturally; however, their levels vary as a result of local influences like diffuse agricultural runoff, inputs to the river from unaccounted for pipes, misconnections and other sources.

**Fig. 8:**
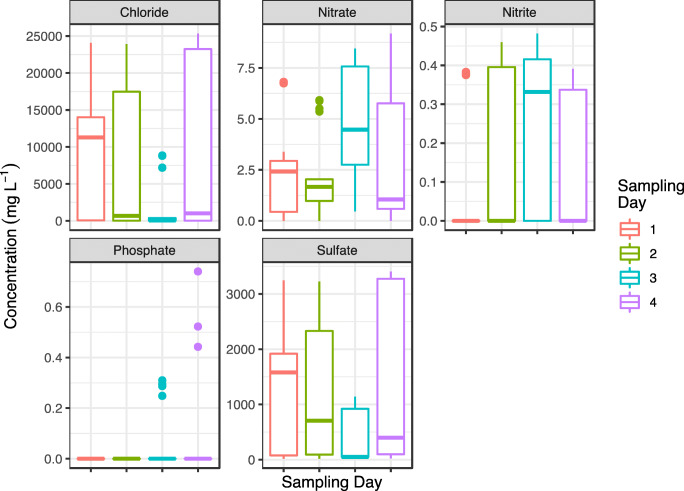
Inorganic anion concentrations (mg L^−1^) for all sampling sites of the four sampling days

**Fig. 9:**
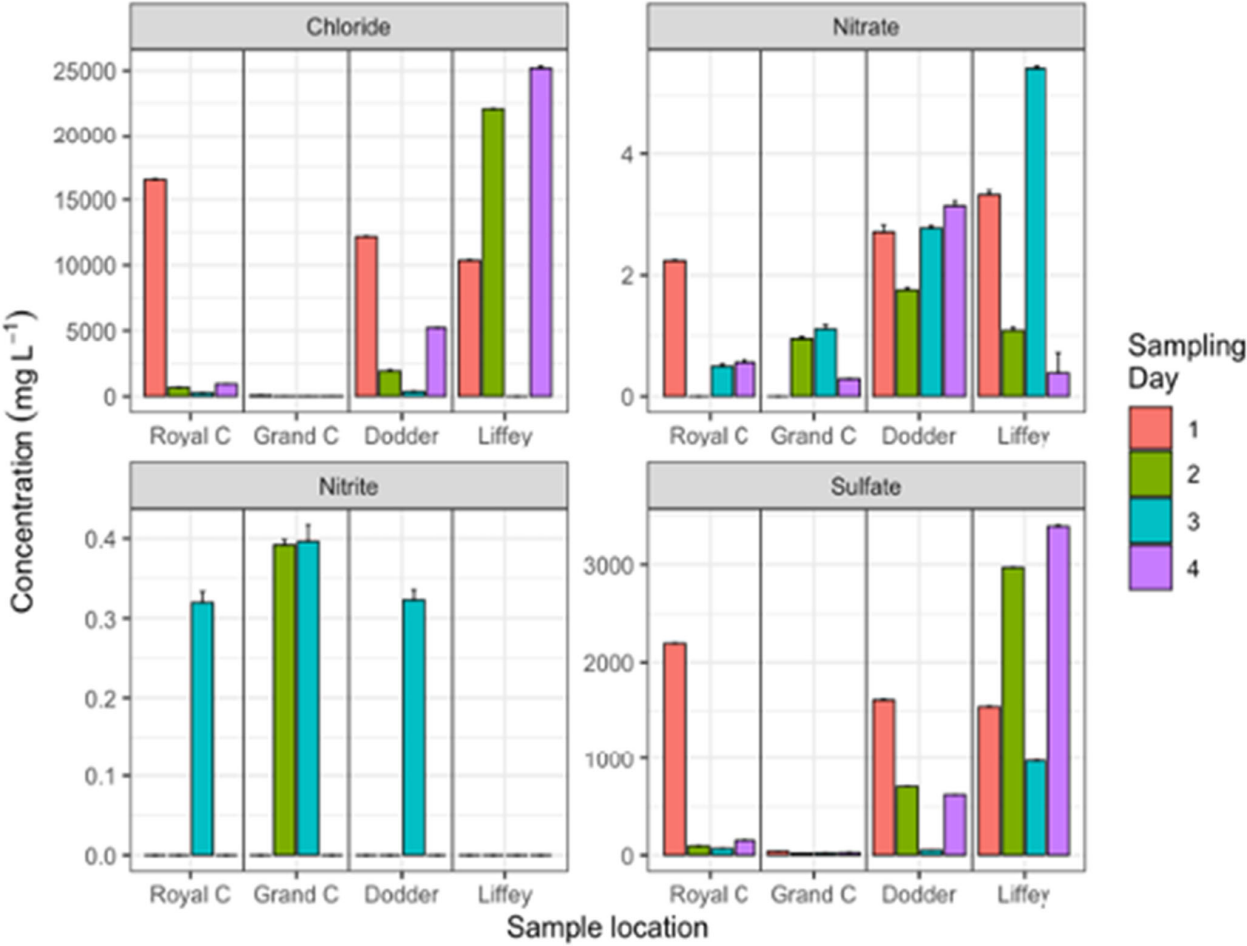
Comparison of inorganic pollutants concentration (mg L^−1^). Levels of species are shown for the Liffey river (Liffey, site 6), the Grand canal (Grand C, site 5), the Royal canal (Royal C, site 7) and the river Dodder (site 4) for each of the four sampling dates. Error bars calculated for n = 3

As can be observed in Fig. 8, phosphate was rarely detected in the river samples. On the other hand, the nitrate content found in the river was higher than the values quantified by other authors in surface water samples in South-eastern Nigeria (Edeogu 2007). It was also observed that there was an increase in the nitrate concentration on Day 3 of sampling. On this day the nitrate concentrations increased downstream as shown in Fig. 9, from the Royal Canal to the river Liffey where the nitrate level was close to 6 mg L^−1^. Comparing the maximum nitrate level in water defined by the World Health Organization of 10 mg L^−1^ (He et al. 2011), to the nitrate levels detected in the river, the found values can be considered as low being variable from sampling day to sampling day. This is a common observation because of the large variation in activities and inputs from the catchment area. The increase of nitrate content on Day 3 suggests a variety of sources of nitrate. The common sources of nitrate to rivers include applications of chemical fertilizer and pesticide in the agricultural areas, domestic sewage, industrial wastewater discharge and plant humus among others (Xue et al. (2016).

In this location there are potential influences from wastewater treatment plant overflows in the area. Inputs of ammonia generated during wastewater treatment can be oxidised and degraded into nitrite and nitrate species. High chloride concentrations were expected in the Liffey sites due to saline water intrusion from the Irish Sea (Vetrimurugan et al. 2013). On the other hand, low levels of chloride were measured in the canal inputs to the Liffey river as these sites were decoupled from the river by lock mechanisms and the primary sources of water are derived from rainfall runoff.

## Conclusions

The impact of rainfall on the occurrence of different chemical classes and their distribution in the Liffey in Dublin was evaluated. It was found that lower levels of chemical contaminants were detected in the days following a rainfall event likely to be arising from dilution and increased flow. On the other hand, it was found that individual tributaries or canals have low impacts on the water quality of the river itself.

The main target organic pollutants found in the samples were phthalates, trichlorophenol, alkylphenols and nitro-aromatic compounds. These chemicals were found in the samples in the range 0.21–2.17 μg L^−1^ with the exception of dimethyl phthalate which was present at higher levels (around 100 μg L^−1^) in certain river samples. Although these compounds have known toxicity and can pose a risk to aquatic ecosystems the detected values were lower than the toxicity levels to several aquatic organism reported in the literature (from 9 to 11,000 μg L^−1^, depending on the compound). Considering the diverse chemical nature and applications of these compounds, the contamination sources of the river can be related to agricultural, industrial activities as well as diffuse urban contributions.

Nitrates were detected in the river in the range from 0.59 to 6.81 mg L^−1^ increasing in concentration from upstream to downstream. These results indicate potential contamination due to agricultural sources as well as influence of wastewater into the river water composition.

The overall results of this study indicate that if we are to meet the 2050 goal of zero pollution, alternatives are required to replace certain chemicals in widespread use. The human and wildlife exposure to the pollutants needs to be further controlled by regular monitoring of the river water quality, taking account of ecological impacts within a water body.

## Supplementary Information

ESM 1 (DOCX 49 kb)

## Data Availability

Original data are available on requests form the corresponding author.
