# Supplementary material for: Assessment of anthropogenic pollution by monitoring occurrence and distribution of chemicals in the river Liffey in Dublin
Source: Environ Sci Pollut Res Int. 2021 May 25;28(38):53754–66. doi: 10.1007/s11356-021-14508-y (PMC8476352; doi:10.1007/s11356-021-14508-y)
Supplement: Supplementary file 1 — (DOCX 49 kb) [file 11356_2021_14508_MOESM1_ESM.docx]

**“Zero Pollution” is currently a pipe dream with ubiquitous phthalate occurrence and other organic and inorganic pollutants in freshwater**

*Rosa Peñalver^1^, Matthew R. Jacobs^2,3^, Susan Hegarty^3,4^, Fiona Regan^2,3*^*

^1^Department of Analytical Chemistry, Faculty of Chemistry, University of Murcia, Murcia, Spain.

^2^School of Chemical Sciences, Dublin City University

^3^DCU Water Institute, Dublin City University, Glasnevin, Dublin, Ireland.

^4^School of History and Geography, Dublin City University, St Patrick’s Campus, Drumcondra, Ireland

*Corresponding author:

Prof. Fiona Regan

School of Chemical Sciences

Dublin City University, Glasnevin, Dublin 9,

Ireland

Tel.: 00 353 1 7005765

e-mail: [fiona.regan@dcu.ie](mailto:fiona.regan@dcu.ie)

| **Table S1**. Selected MS ions, detection and quantification limits (µg L^-1^) for all the compounds | | | | |
| --- | --- | --- | --- | --- |
| Compound (CAS number) | Ion 1 | Ion 2 | LOD (µg L^-1^) | LOQ (µg L^-1^) |
| Phenol (108-95-2) | 94 | 66 | 0,01 | 0.17 |
| Aniline (62-53-3) | 93 | 66 | 0.04 | 0.13 |
| Bis(2-chloroethyl)ether (111-44-4) | 93 | 63 | 0.04 | 0.13 |
| 2-chlorophenol (95-57-8) | 128 | 64 | 0.05 | 0.17 |
| 1,3-dichlorobenzene (541-73-1) | 146 | 148 | 0.025 | 0.083 |
| 1,4-dichlorobenzene (106-46-7) | 146 | 148 | 0.025 | 0.083 |
| benzyl alcohol (100-51-6) | 108 | 79 | 0.035 | 0.12 |
| 1,2-dichlorobenzene (95-50-1) | 146 | 148 | 0.045 | 0.15 |
| 2-methylphenol (95-48-7) | 107 | 108 | 0.045 | 0.15 |
| 2,2'oxybis(1-chloropropane) (108-60-1) | 121 | 123 | 0.105 | 0.35 |
| 3-methylphenol (108-39-4) | 107 | 108 | 0.035 | 0.12 |
| 4-methylphenol (106-44-5) | 107 | 108 | 0.085 | 0.28 |
| N-nitroso-di-n-propylamine (621-64-7) | 130 | 70 | 0.04 | 0.13 |
| Hexachloroethane (67-72-1) | 117 | 201 | 0.075 | 0.25 |
| Nitrobenzene (98-95-3) | 77 | 123 | 0.07 | 0.23 |
| Isophorone (78-59-1) | 82 | 138 | 0.05 | 0.17 |
| 2-nitrophenol (88-75-5) | 139 | 109 | 0.045 | 0.15 |
| 2,4-dimethylphenol (105-67-9) | 122 | 107 | 0.025 | 0.083 |
| Bis(2-chloroethoxy)methane (111-91-1) | 93 | 95 | 0.05 | 0.17 |
| 2,4-dichlorophenol (120-83-2) | 162 | 164 | 0.03 | 0.1 |
| 1,2,4-trichlorobenzene (120-82-1) | 180 | 182 | 0.045 | 0.15 |
| Naphthalene (91-20-3) | 128 | 129 | 0.025 | 0.083 |
| 4-chloroaniline (106-47-8) | 127 | 129 | 0.075 | 0.25 |
| Hexachlorobutadiene (87-68-3) | 225 | 223 | 0.1 | 0.33 |
| 4-chloro-3-methylphenol (59-50-7) | 107 | 144 | 0.025 | 0.083 |
| 2-methylnaphthalene (91-57-6) | 142 | 141 | 0.025 | 0.083 |
| 1-methylnaphthalene (90-12-0) | 142 | 141 | 0.025 | 0.083 |
| Hexachlorocyclopentadiene (77-47-4) | 235 | 237 | 0.15 | 0.48 |
| 2,4,6-trichlorophenol (88-06-2) | 196 | 198 | 0.07 | 0.23 |
| 2,4,5-trichlorophenol (95-95-4) | 196 | 198 | 0.08 | 0.27 |
| 2-chloronaphthalene (91-58-7) | 162 | 127 | 0.025 | 0.083 |
| 2-nitroaniline (88-74-4) | 65 | 92 | 0.03 | 0.1 |
| 1,4-dinitrobenzene (100-25-4) | 168 | 75 | 0.225 | 0.75 |
| Dimethylphthalate (131-11-3) | 163 | 194 | 0.14 | 0.4 |
| 41,3-dinitrobenzene (99-65-0) | 168 | 76 | 0.025 | 0.083 |
| 2,6-dinitrotoluene (606-20-2) | 165 | 50 | 0.19 | 0.63 |
| 1,2-dinitrobenzene (528-29-0) | 168 | 50 | 0.24 | 0.78 |
| Acenaphthylene (208-96-8) | 152 | 151 | 0.125 | 0.42 |
| 3-nitroaniline (99-09-2) | 138 | 108 | 0.025 | 0.083 |
| Acenaphthene (83-32-9) | 154 | 153 | 0.085 | 0.28 |
| 2,4-dinitrophenol (51-28-5) | 184 | 63 | 0.025 | 0.083 |
| 4-nitrophenol (100-02-7) | 139 | 109 | 0.73 | 2.43 |
| 2,4-dinitrotoluene (121-14-2) | 165 | 63 | 0.5 | 1.67 |
| Dibenzofuran (132-64-9) | 168 | 139 | 0.165 | 0.55 |
| 2,3,4,6-tetrachlorophenol (58-90-2) | 232 | 131 | 0.025 | 0.083 |
| 2,3,5,6-tetrachlorophenol (935-95-5) | 232 | 131 | 0.2 | 0.67 |
| Diethylphthalate (84-66-2) | 149 | 177 | 0.175 | 0.58 |
| 4-chlorophenyl phenyl ether (7005-72-3) | 204 | 206 | 0.025 | 0.083 |
| Florene (86-73-7) | 166 | 165 | 0.05 | 0.17 |
| 4-nitroaniline (100-01-6) | 138 | 65 | 0.025 | 0.083 |
| 4,6-dinitro-2-methylphenol (534-52-1) | 198 | 105 | 0.275 | 0.920 |
| Diphenylamine (122-39-4)** | 169 | 168 | 0.035 | 0.12 |
| Azobenzene (103-33-3)* | 182 | 152 | 0.1 | 0.33 |
| 4-bromophenyl phenyl ether (101-55-3) | 248 | 250 | 0.04 | 0.13 |
| Hexachlorobenzene (118-74-1) | 284 | 142 | 0.04 | 0.13 |
| Pentachlorophenol (87-86-5) | 266 | 264 | 0.275 | 0.92 |
| Phenanthrene (85-01-8) | 178 | 179 | 0.025 | 0.083 |
| Anthracene (120-12-7) | 178 | 179 | 0.025 | 0.083 |
| Carbazole (86-74-8) | 167 | 166 | 0.025 | 0.083 |
| Di-n-butylphthalate (84-74-2) | 149 | 150 | 0.025 | 0.083 |
| Fluoranthene (206-44-0) | 202 | 101 | 0.025 | 0.083 |
| Pyrene (129-00-0) | 202 | 200 | 0.025 | 0.083 |
| Benzyl butyl phthalate (85-68-7) | 149 | 206 | 0.03 | 0.1 |
| Bis(2-ethylhexyl)adipate (103-23-1) | 147 | 129 | 0.15 | 0.5 |
| Bis(2-ethylhexyl)phthalate (117-81-7) | 149 | 167 | 0.18 | 0.6 |
| benz(a)anthracene (56-55-3) | 228 | 229 | 0.065 | 0.22 |
| Chrysene (218-01-9) | 228 | 226 | 0.025 | 0.083 |
| Di-n-octyl phthalate (117-84-0) | 149 | 167 | 0.07 | 0.23 |
| Benzo(b)fluoranthene (205-99-2) | 252 | 253 | 0.09 | 0.3 |
| Benzo(k)fluoranthene (207-08-9) | 252 | 253 | 0.085 | 0.28 |
| Benzo(a)pyrene (50-32-8) | 252 | 253 | 0.215 | 0.72 |
| Indeno(1,2,3-cd)pyrene (193-39-5) | 276 | 138 | 0.355 | 1.78 |
| Dibenz(a,h)anthracene (53-70-3) | 278 | 139 | 0.29 | 0.97 |
| Benzo(g,h,i)perylene (191-24-2) | 276 | 138 | 0.28 | 0.93 |

| **Table S2.** Group classification of the targeted compounds | | |
| --- | --- | --- |
| Compound | Groups (Figures 4,5 and 6) | Groups (Figure 7) |
| Di-n-butylphthalate | Phthalates | Phthalates |
| Di-n-octyl phthalate | Phthalates | Phthalates |
| Diethylphthalate | Phthalates | Phthalates |
| Dimethylphthalate | Phthalates | Phthalates |
| 2-nitroaniline | Aromatic compounds | Nitro compounds |
| Benzyl butyl phthalate | Phthalates | Phthalates |
| 3-nitroaniline | Aromatic compounds | Nitro compounds |
| 1,2-dinitrobenzene | Aromatic compounds | Nitro compounds |
| Phenol | Aromatic compounds | Phenolic compounds |
| 2,4-dimethylphenol | Aromatic compounds | Phenolic compounds |
| 2-methylphenol | Aromatic compounds | Phenolic compounds |
| Carbazole | Aromatic compounds | Aromatic compounds |
| 2,6-dinitrotoluene | Aromatic compounds | Nitro compounds |
| 4-nitroaniline | Aromatic compounds | Nitro compounds |
| 3/4-methylphenol | Aromatic compounds | Phenolic compounds |
| 2-nitrophenol | Aromatic compounds | Nitro compounds |
| 1,3-dinitrobenzene | Aromatic compounds | Nitro compounds |
| Isophorone | Aliphatic compounds | Aliphatic compounds |
| 1,4-dinitrobenzene | Aromatic compounds | Nitro compound |
| Benzyl alcohol | Aromatic compounds | Phenolic compounds |
| Bis(2-chloroethoxy)methane | Aliphatic compounds | Aliphatic compounds |
| Diphenylamine | Aromatic compounds | Phenolic compounds |
| N-nitroso-di-n-propylamine | Aliphatic compounds | Aliphatic compounds |
| 2,4-dichlorophenol | Aromatic compounds | Phenolic compounds |
| 4-chloroaniline | Aromatic compounds | Halogenated compounds |
| 2-chlorophenol | Aromatic compounds | Halogenated compounds |
| 2,4-dinitrotoluene | Aromatic compounds | Nitro compounds |
| Bis(2-chloroethyl)ether | Aliphatic compounds | Halogenated compounds |
| Azobenzene | Aromatic compounds | Aromatic compounds |
| 2,4,6-trichlorophenol | Aromatic compounds | Halogenated compound |
| 4-chloro-3-methylphenol | Aromatic compounds | Halogenated compounds |
| Aniline | Aromatic compounds | Aromatic compounds |
| Nitrobenzene | Aromatic compounds | Nitro compounds |
| Fluorene | Aromatic compounds | Aromatic compounds |


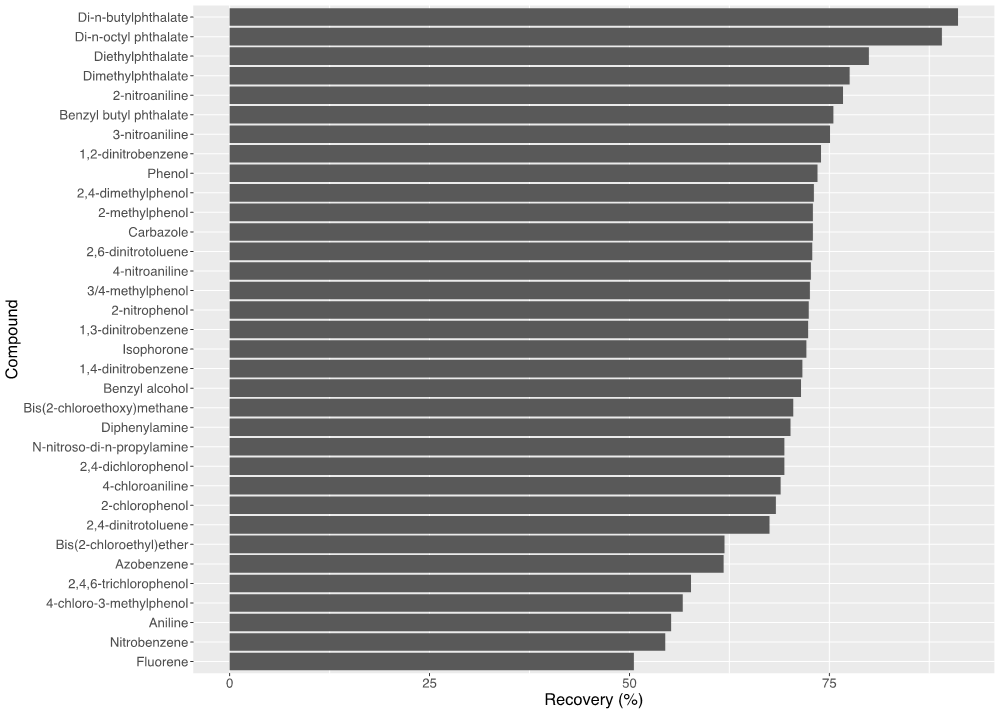


**Figure S1**. Recovery data for compounds with recovery over 50%.
